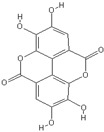# Olive Oil Effects on Colorectal Cancer

**DOI:** 10.3390/nu11010032

**Published:** 2018-12-23

**Authors:** Antonio Maria Borzì, Antonio Biondi, Francesco Basile, Salvatore Luca, Enzo Saretto Dante Vicari, Marco Vacante

**Affiliations:** 1 Department of Clinical and Experimental Medicine, Postgraduate Specialization School in Geriatrics, University of Catania, AOU Policlinico, 95123 Catania, Italy; antoniomaria.borzi@gmail.com (A.M.B.); enzodante@email.it (E.S.D.V.); 2 Department of General Surgery and Medical-Surgical Specialties, University of Catania, Via S. Sofia 78, 95123 Catania, Italy; abiondi@unict.it (A.B.); fbasile@unict.it (F.B.); salvatoreluca@virgilio.it (S.L.)

**Keywords:** olive oil, colorectal cancer, phenols, microbiota, antioxidants, inflammation

## Abstract

Colorectal cancer is the fourth cause of cancer-related death worldwide. A Mediterranean diet showed protective action against colorectal cancer due to the intake of different substances. Olive oil is a fundamental component of the Mediterranean diet. Olive oil is rich in high-value health compounds (such as monounsaturated free fatty acids, squalene, phytosterols, and phenols). Phenolic compounds exert favourable effects on free radicals, inflammation, gut microbiota, and carcinogenesis. The interaction between gut microbiota and olive oil consumption could modulate colonic microbial composition or activity, with a possible role in cancer prevention. Gut microbiota is able to degrade some substances found in olive oil, producing active metabolites with chemopreventive action. Further clinical research is needed to clarify the beneficial effects of olive oil and its components. A better knowledge of the compounds found in olive oil could lead to the development of nutritional supplements or chemotherapeutic agents with a potential in the prevention and treatment of colorectal cancer.

## 1. Introduction

Colorectal cancer (CRC) is the third most common cancer per incidence worldwide, the third in men and the second in women (746,000 and 614,000 cases, 10.0% and 9.2% of the total respectively). More than half of the cases of CRC occur in industrialized regions of the world [1]. CRC patients show a 5-year survival of 64% in the United States, with a similar number of deaths in the two sex [2]. Following a healthy diet could be a measure of primary prevention for CRC as dietary habits are estimated to contribute to about 50% of CRC cases [3,4]. There is growing evidence that the adoption of the Mediterranean diet (MD) could represent a protective factor against the onset of various types of cancer, including CRC [5]. The anti-tumor effects of the MD are largely due to the combination of antioxidant elements, fiber and polyunsaturated fats [6,7]. A number of studies showed the beneficial effects of MD, mainly regarding the lower rate of cardiovascular diseases, atherosclerosis, and some types of tumors (i.e., intestinal, breast, and prostate cancers) [8]. A meta-analysis of eight studies showed a significant inverse association between adherence to MD and incidence of CRC, with a 17% risk reduction for CRC when comparing the highest versus lowest MD categories [9].

Olive oil (OO) (*Olea europaea, Oleaceae*) is a fundamental component of the MD; it is a mix of fatty acids such as oleic and linoleic acid, secoiridoids (oleuropein and oleocanthal), simple phenols (tyrosol and hydroxytyrosol), lignans (pinoresinol), flavonoids (apigenin), hydrocarbons (squalene), triterpenes (maslinic acid), and phytosterols (β-sitosterol). The chemical composition of OO depends on the extraction method used to obtain oil from the olives. Olives are crushed and then the oil is separated from the fruit pulp applying high pressure. Additional processes include extrusion, post-pression or re-pression, with or without the use of hot water. The OO obtained through additional methods shows a stronger colour, weaker flavour, and a higher concentration of free fatty acids [10,11]. Virgin olive oils (VOOs) are extracted from the olives exclusively by mechanical or other physical means under conditions that does not alter the oil. Extra-VOOs (EVOOs) are obtained from once cold-pressed unfermented olives, and contain a low percentage of free fatty acids (<1%) and the highest phenols levels [12].

During OO extraction, a large amount of waste water (olive mill wastewater, OMWW) is produced to separate the oil from the paste. OMWW is a pollutant, but it can be used to produce an extract rich in polyphenols [13]. The phenolic fraction in OO may range from 50 to 800 mg/kg, according to variables such as the climatic conditions, the type of cultivar, the maturity of the drupes at harvesting, and the methods used to produce the different types of OO: EVOO, VOO, or OO [14]. A large body of epidemiological evidence suggests favourable effects of compounds found in OO, especially phenols, on free radicals, inflammation, gut microbiota and carcinogenesis [15,16] (Table 1).

The concentration of many OO substances used in in vitro studies, which showed anti-tumor properties, cannot be achieved in vivo with OO or supplement diet [17]. Polyphenols showed a reduced bioavailability due both to incomplete gut absorption and to quick biotransformation and urinary excretion [18]. However, there is a lack of information concerning the bioavailability of most OO polyphenols, even if intensive research has been conducted in the last few years [19].

OO shows less chemico-physical modifications with heating as compared to other vegetable oils. A study showed that OO, subjected to heating at 180 °C for 36 h, preserved most of its substances and nutritional characteristics [20]. Polycyclic aromatic hydrocarbons (PAHs) and heterocyclic amines (HCAs) are potentially mutagenic substances produced in cooking oils when repeatedly heated, at high temperatures [21,22,23]. PAHs and HCAs could be involved in CRC carcinogenesis [24,25]. However, the incidence of CRC did not seem to increase in consumers of food fried in OO [23]. OO is preferable for its properties and substances to other vegetable oils for cooking, if used once and heated under 180 °C [26].

The aim of this review was to summarize the current knowledge on the beneficial effects of OO and its components, independently of the extraction method, as a preventive or potential therapeutic agent for the treatment of CRC.

## 2. Effects of Olive Oil Phenols on CRC

Phenols are the most studied components in OO with recognized antitumor properties [45]. The unsaponificable fraction of OO contains the phenolic compounds, while the saponificable fraction is rich in monounsaturated fatty acids (MUFA) (i.e., oleic acid) [46]. Phenolic compounds can be divided into three groups: simple phenols (i.e., tyrosol, hydroxytyrosol or 3,4-dihydroxyphenylethanol), phenolic acids (i.e., caffeic acid), and flavonoids (i.e., quercetin). The most important complex phenols are tyrosol and hydroxytyrosol esters, oleuropein, and its aglycone [47]. Hydroxytyrosol, tyrosol and oleuropein, have similar structure and are found at high levels in OO [48] (Table 2). There is evidence that OO polyphenols could reduce oxidative damage to cellular DNA thus decreasing the development of CRC. High levels of antioxidant polyphenols (i.e., hydroxytyrosol, oleuropein) may reduce the amount of potentially carcinogenic products of lipid peroxidation upon OO storage, may lead to positive epigenetic changes [49] and miRNA expression pattern, thus decreasing the risk of CRC development [50,51]. Oxidative stress is due to an imbalance between the oxidant and the antioxidant systems of the organism, in favour of the oxidants. Oxidation is mainly caused by free radicals and the related reactive oxygen species (ROS). The free radicals are able to damage fatty acids and to provoke a chain reaction called lipid peroxidation, which may lead to loss of membrane function and integrity and finally to apoptosis and necrosis [52,53].

Many studies showed that hydroxytyrosol (HT) has significant anti-inflammatory [54] and antitumor effects [55]. Other beneficial properties of HT include improvement of endothelial cell function [56], protective effect on liver steatosis [57], and neuroprotective effects [58]. The European Food Safety Authority (EFSA) Panel on Dietetic Products, Nutrition and Allergies (NDA) indicated HT as a polyphenol able to protect low density lipoproteins (LDL) against oxidative modifications, and recommended a daily consumption of at least 5 mg of HT and its derivatives (i.e., oleuropein complex and tyrosol) in OO [59].

A study by Mateos et al. demonstrated that OO HT acetate may exert antitumor activity on human colon adenocarcinoma cells, affecting the transcription of genes involved in programmed cell death (BNIP3, BNIP3L, PDCD4, and ATF3), and activating caspase-3. HT acetate also may enhance carcinogen detoxification, upregulating xenobiotic metabolizing enzymes UGT1A10 and CYP1A1 [28]. Other studies indicated that HT could be able to inhibit cancer proliferation inducing cell cycle arrest and apoptosis in different tumors, such as cholangiocarcinoma [60].

HT may downregulate epidermal growth factor (EGFR) expression and inhibit cell cycle progression in colon cancer cells, similarly to cetuximab, a monoclonal antibody against EGFR [29]. A recent study by Terzuoli et al. suggested a potential benefit of a controlled diet containing OO, during cetuximab chemotherapy [30]. HT could improve the effects of EGFR inhibitors acting as a useful therapeutic agent in patients with colon cancer [30]. Interestingly, other polyphenols, such as curcumin and resveratrol, showed therapeutic benefits on CRC when combined with chemotherapeutic agents [61,62].

Purified extracts from OMWW, rich in HT, showed anti-angiogenic and chemopreventive effects both in vivo and in vitro on CT-26 CRC cell line [13].

Rossi et al. investigated the properties of A009, which is a purified polyphenol enriched extract from OMWW. A009 showed strong anti-angiogenic effects both in vitro and in vivo. The inhibition of angiogenesis by A009 was more potent when compared to the effect of the same concentrations of HT. The A009 extract was also effective on the inhibition of endothelial cell proliferation, migration and invasion [27]. These findings shed a light on possible future applications of OMWW for cancer preventive strategies, recovering agricultural waste products.

Oleuropein (an ester of hydroxytyrosol with β-glucosylated elenolic acid) is the major phenolic compound in olive cultivars and is responsible for the bitter and pungent taste of olives. The concentrations of oleuropein may range from around 140 mg g^−1^ in dry young olives [63] to 60–90 mg g^−1^ in dry leaves [64]. Oleuropein showed many pharmacological activities and beneficial effects on cancer, oxidation, inflammation, atherogenesis, viral and bacterial infections, dyslipidaemia and had also hypoglycemic properties [48,65]. A study by Hamdi and Castellon pointed out the antitumor effect of oleuropein, which was able to directly disrupt actin filaments in cells and in a cell-free assay, and to inhibit proliferation and migration of advanced-grade tumor cell lines (including LoVo, colorectal adenocarcinoma cells) in a dose-responsive way. Also, oral administration of oleuropein to mice that developed spontaneous tumors was followed by tumor regression in 9–12 days [31].

Another study evaluated the anticancer effects of oleuropein on HT-29 human colon adenocarcinoma cells as compared to HT, its hydrolysis product. The results indicated that oleuropein caused significant changes in cell cycle analysis, limiting cell growth and inducing apoptosis. The effects of oleuropein were mediated by p53 pathway activation, adapting the HIF-1α (hypoxia-inducible factor 1-α) protein response to hypoxia. On the other hand, HT caused a significant upregulation of peroxisome proliferator-activated receptor gamma (PPARγ), which failed in oleuropein, and represented the main antitumor mechanism exerted by HT [32,66].

Giner et al. pointed out that oleuropein as a dietary supplementation could represent a promising protective agent against colitis-associated CRC. Actually, the authors showed that the beneficial effects of oleuropein in a model of azoxymethane (AOM)/Dextran sulfate sodium (DSS)-induced CRC in C57BL/6 mice, included the reduction of intestinal IFN-γ, TNF-α, IL-6, and IL-17A levels, and also the decrease of cyclooxygenase-2, Bax and proliferating cell nuclear antigen protein expression. Also, it has been observed a marked down-regulation of CRC-related pathways such as Wnt/β-catenin, phosphatidylinositol-3-kinase (P3IK)/Akt, nuclear factor-κB (NF-κB), and signal transducer and activators of transcription (STAT)3 [33,67].

OO components could prevent cancer development down-regulating the angiogenesis signalling pathways. The expression of VEGF and other angiogenic factors is enhanced in different cancers. Treatment with anti-angiogenic drugs showed favourable effects in cancer, but it is associated with adverse side effects [68]. In tumor cells, oleuropein inhibits the pathways involved in proliferation and migration, exerting antioxidant and anti-angiogenic effects with a dose-related way [69,70]. Anti-tumor effects of oleocanthal include cell apoptosis by activating caspase-3 and poly-adenosine diphosphate-ribose polymerase, phosphorylates p53 (Ser15), and also the disruption of DNA in HT-29 cells derived from human colon adenocarcinoma [34]. A study by Margarucci et al. showed a statistically significant reduction of two heat shock proteins 90 (Hsp90), Akt and Cdk4, as a consequence of the inhibition of chaperone activity by oleocanthal [71]. In this context, it is noteworthy that inhibition of Hsp90 and other Hsps has recently emerged as a novel therapeutic strategy for cancer treatment [72].

## 3. Anti-Inflammatory, Immunomodulatory and Other Anticancer Properties of Olive Oil

Inflammatory response could be modulated by OO polyphenols, which are able to inhibit NF-κB as demonstrated in both in vitro and in vivo studies. The inhibition of NF-κB results in low expression of IL-6, IL-8, IL-1β and COX-2, with a consequent creation of a microenvironment that hinders cancer growth [73,74]. Beauchamp et al. observed that decarboxy methyl ligstroside aglycone (also known as oleocanthal) possesses an anti-inflammatory action similar to that of ibuprofen. In fact, both molecules are able to inhibit cyclooxygenase (COX) enzymes involved in the biosynthesis of prostaglandins [75]. The anticarcinogenic and antithrombotic effects of COX inhibitors, such as ibuprofen and aspirin, are well known [76,77]. It is possible that the administration of oleocanthal may help to reduce the development of inflammatory bowel diseases (IBD) (ulcerative colitis and Crohn’s disease), and in turn to decrease the risk of CRC [78,79]. IBD represent a major risk factor for the development of CRC [80,81]. Although CRC occurs in a small number of patients with IBD (1%), it shows a high mortality and is responsible for 20% of IBD-related mortality [82].

A study conducted in vivo on rats with azoxymethane induced CRC suggested the chemopreventive effect of OO against colon carcinogenesis. The antitumor activity seems to be related to the modulation in colonic mucosa of arachidonic acid metabolism and prostaglandin E2 synthesis, exerted by n9 and n3 fatty acids (oleic acid and eicosapentaenoic acid respectively) present in OO [35]. The phenolic compounds in OO, besides to anti-inflammatory properties, showed also immunomodulatory effects. The immunomodulatory properties could reduce chronic inflammation in IBD and also in other immune-mediated pathologies, such as multiple sclerosis, psoriasis, rheumatoid arthritis, systemic lupus erythematosus and inflammatory bowel diseases [83]. The primarily involved cells in the autoimmune and inflammatory responses are T lymphocytes and antigen presenting cells (APCs), which are B cells monocyte/macrophages and dendritic cells [84]. Raised levels of inflammatory cytokines (i.e., TNF-alpha, IL-8, IL-10, IL-6, IL-17) and activation of innate adaptive immune cells are involved in the pathogenesis and evolution of IBD. On this basis, cytokine pathways modulating drugs could be used in IBD, although side effects and symptoms recurrence are common [85]. Dietary OO phenols seem to change clinico-pathological history in IBD, due to their anti-inflammatory properties [86].

Apigenin belongs to the subclass of flavonoids in OO and has been widely used in traditional Chinese medicine for centuries. Apigenin has been demonstrated to show anti-tumor properties in colorectal, liver, breast, lung, and prostate cancer, with low toxicity and no mutagenic activity [87]. Apigenin showed dose-dependent activity on proliferation, migration and invasion in CRC, modulating signaling pathways such as JAK/STAT, PI3K/AKT, NF-κB, MAPK/ERK, and Wnt/β-catenin pathways [36,87]. It has been observed that apigenin had a synergistic action with ABT-263, a BH-3 mimetic, on CRC cells apoptosis by blocking functions of Bcl-2 family proteins [37,88]. Based on these findings, apigenin could be used as dietary supplement or in combination with chemotherapeutic drugs for CRC treatment [89].

Luteolin is another natural flavonoid contained in glycosylated form in OO. Glycosylated luteolin is hydrolyzed to free luteolin during intestinal absorption. With other phenolic antioxidants, luteolin demonstrated different beneficial properties on inflammation, oxidation, and cancers [90]. Luteolin modulated the G2/M cell cycle arrest and caused apoptosis in CRC cells [38]. Also, luteolin blocked the cell diversion to CRC by epigenetically activating the nuclear factor erythroid 2-related factor 2 (Nrf2)/antioxidant-responsive element (ARE) pathway [39].

Maslinic acid (MA) is a triterpene found at high levels in the waxy skin of olives. A study by Sànchez-Quesada et al. demonstrated that MA modulated the inflammation process by stimulating the production of, IL-1α, IL-1β and IL-8, increased IFN-γ level, which led to M1 polarization, and did not affect the levels of NF-κB or nitric oxide (NO). These findings suggested that MA could prevent chronic inflammatory response, which is involved in carcinogenesis [91]. MA was also demonstrated to induce apoptosis via the intrinsic apoptotic pathway associated with mitochondria in HT29 colon cancer cells [41]. Another study showed that MA may induce apoptotic cell death via the extrinsic apoptotic pathway in Caco-2 colon cancer cells, leading to the cleavage of caspases -8 and -3, and to an increase of t-Bid levels, in a dose-dependent way [40]. Given the possibility to activate both apoptotic pathways, MA could represent a natural compound with chemotherapeutic or chemopreventive actions in CRC.

β-sitosterol, a phytosterol found in OO, inhibited significantly the growth of COLO 320 DM cells, in a dose-dependent way, caused apoptosis by scavenging ROS, and suppressed the expression of beta-catenin and proliferating cell nuclear antigen (PCNA) in human colon cancer cells [42]. A case-control study carried out in a Chinese population, showed that the consumption of phytosterols, including β-sitosterol, campesterol and campestanol was associated with a reduction of CRC risk making it a potential anticancer drug for colon carcinogenesis [92].

## 4. Effects of Olive Oil Fatty Acids on CRC

The fatty acids in OO are principally represented by oleic acid followed by palmitic and linoleic acids. The effects of OO-derived MUFAs on CRC have not been widely studied, but there is evidence that they could have either no role or a protective role on the carcinogenesis in this tumor [93,94]. The high content of oleic acid gives to OO more resistance to oxidation than the polyunsatured fatty acids (PUFAs) [95]. Some studies highlighted that oleic acid, linoleic acid and squalene could have a tumor-inhibiting role [96]. The beneficial effects derived from MUFAs seems to be due principally to oleic acid [97]. In fact, the intake of oleic and linoleic acid resulted in the induction of apoptosis and cell differentiation, mediated by an early downregulation of COX-2 followed by a reduction in Bcl-2 expression [98]. Butler et al. observed a significant inverse correlation between colon cancer risk and higher plasmatic concentrations of oleic, α-linolenic and linoleic acids in the Singapore Chinese Health Study, whereas a statistically significant positive association with colon cancer was observed for arachidonic acid [99].

Squalene, an acyclic hydrocarbon, could inhibit the catalytic activity of beta-hydroxy-beta-methylglutaryl-CoA reductase, leading to a reduction of farnesyl pyrophosphate availability for prenylation of the ras oncogene, thus relocating this oncogene to cell membranes [96].

## 5. Effects of Olive Oil on Gut Microbiota

The gut microbiota is represented by a composite and dynamic population of microorganisms found in the human gastrointestinal tract, which strongly influence the host as regards homeostasis and diseases [100]. Some studies showed the fundamental impact of diet in shaping the gut microbiota across the lifetime [101]. There is growing evidence that the gut microbiota may play a crucial role in the development and evolution of gastrointestinal malignancy [102,103,104]. The interaction between mucosal inflammation, oxidative stress and gut microbiota may influence the pathogenesis of CRC in patients with IBD [105]. OO consumption is proven to influence the composition of intestinal microbiota; some studies highlighted a significant modulation effect of dietary polyphenols on the colonic microbial composition or activity, with a possible role on cancer prevention [106,107]. The intake of OO polyphenols may favour a healthy gut microbiota, increasing bifidobacteria and the amount of intestinal IgA-coated bacteria [83]. However, the mechanisms underlying the association between gut microbiota and carcinogenesis are not fully established.

A study by Miene et al. analyzed the effects of 3,4-dihydroxyphenylacetic acid and 3-(3,4-dihydroxyphenyl)-propionic acid, which are metabolites of quercetin and chlorogenic acid/caffeic acid, respectively, in human colon adenoma cells LT97. The results showed an enhancement of glutathione S-transferase T2 (GSTT2) expression and a decrease of COX-2 that could explain the chemopreventive action of polyphenol metabolites after intestinal degradation [43]. A study by Kang et al. observed that caffeic acid may inhibit colon cancer metastasis and neoplastic cell transformation by suppressing mitogen-activated MEK1 and TOPK activities [108]. Polyphenols and flavons found in OO (i.e., epigallocatechin-3-gallate, and quercetin) could have anticancer properties mediated by gut biotransformation [109,110,111].

Stoneham et al. hypothesized that OO may affect secondary bile acid patterns in the bowel and, in turn, modulate polyamine metabolism in colonic cells decreasing the progression sequence from normal mucosa to adenoma and carcinoma [112]. Bile acids promote the growth and activity of 7α-dehydroxylating bacteria, which convert primary into secondary bile acids with tumorigenic properties, principally deoxycholic acid (DCA). Bile acids are able to modify the intestinal microbiota composition due to their antimicrobial activity. Based on these observations, dietary intervention, including intake of OO, could reduce CRC risk through its effects on colonic microbial metabolism [113].

Ellagic acid is another polyphenol found in OO, that showed a number of biological properties such as antioxidant and cancer protective effects on different tumour cell lines, for example Caco-2 colon, Hs 578T breast, MCF-7 breast, and DU 145 human prostatic cancer cells, without any toxicity on normal human lung fibroblasts [44]. Other mechanisms could to be implicated in cancer prevention through the effects of dietary intervention on the gut microbiota, such as the concentrations of polyphenols and other compounds effectively introduced with diet.

## 6. Conclusions

OO contains a variety of beneficial substances that could be helpful for the prevention or the possible treatment of CRC. The large body of evidence supports the chemotherapeutic potential of substances found in OO against CRC, acting on different sides, such as inflammation, oxidative damage, and even epigenetic modulation. It is noteworthy that waste products from OO extraction could be used to produce food supplements with potential effects on cancer prevention. There are few studies reviewing the association between intestinal microbial composition and function and CRC occurrence. However, the strict interaction between OO polyphenols and human microbiota seems to have a beneficial effect on CRC. In conclusion, the consumption of OO should be suggested in a healthy diet instead of other types of oils. The main limitations of existing scientific literature come from the difficult evaluation of a single nutrient in a complex diet, such as the MD. Moreover, several studies have been conducted on animals and properties of OO have been assessed principally by in vitro models. Further studies and clinical trials are needed to better investigate the beneficial effects of OO and its components in humans.

## Figures and Tables

**Table 1:** Principal intervention studies on the effects of olive oil substances on colorectal cancer.

Study (Year)	Design (Cancer Type)	Intervention and Substances Supplementation	Dosage	Effects
Bassani et al. (2016) [13]	in vitro (CT-26 CRC cell line) in vivo (syngenic BalbC mice with CT-26 CRC cell line)	Purified extracts from OMWW rich in HT	HT: 2.7–5.72 g/L	↓VEGF, ↓IL-8 ↓cell migration and invasion ↓tumor cell growth ↓cell adhesion
Rossi et al. (2015) [27]	in vitro (human umbilical vein endothelial cells) in vivo (matrigel sponge assay)	A009 (phenol rich purified extract from OMWW)	1/1000 to 1/250 dilution HT: 2.7–5.72 g/L	Anti-angiogenetic and pro-apoptotic effects ↓endothelial cell proliferation, migration and invasion
Mateos et al. (2013) [28]	in vitro (adenocarcinoma Caco-2/TC7 cells)	HT-acetate	5–50 μM	↓cell proliferation Cell cycle arrest (↑p21 and CCNG2, ↓ CCNB1) Apoptosis (↑BNIP3, BNIP3L, PDCD4, ATF3 and caspase-3) ↑ carcinogen detoxification (CYP1A1 and UGT1A10)
Terzuoli et al. (2016) [29]	in vitro (human colorectal adenocarcinoma cells HT-29, CaCo2, and WiDr) in vivo (mice with HT-29 xenografts)	HT	in vitro100 μM in vivo 10 mg/Kg (200 μL)	↓ tumor cell growth (↑EGFR degradation: EGFR phosphorylation at pY1045 and ↑ Cbl activity with EGFR ubiquitination)
Terzuoli et al. (2017) [30]	in vitro (HT-29 and WiDr cells)	HT-cetuximab combination	HT (10 μM) with cetuximab (1 μg/mL)	↓ tumor cell growth (cell cycle blockade at G2/M phase) ↓cyclins B, D1, and E, and CDK2, CDK4, and CDK6 ↑ CDK inhibitors p21 and p27
Hamdi and Castellon (2005) [31]	in vitro (TF-1a; 786-O, T-47D, RPMI-7951, and colon cancer LoVo) in vivo Swiss albino mice with spontaneous soft tissue sarcomas	Oleuropein	in vitro 0.005–0.1%: in vivo 1% in drinking water	↓ cell proliferation, motility and invasion ↑actin filament disruption Dramatic tumor regression in mice
Cárdeno et al. (2013) [32]	in vitro HT-29 human colon adenocarcinoma cells	Oleuropein	200–400 μM	↓ cell proliferation (p53 pathway activation and ↓HIF-1α)
Giner et al. (2016) [33]	in vivo model of azoxymethane/Dextran sulfate sodium-induced CRC in C57BL/6 mice	Oleuropein	50–100 mg/Kg	Chemoprevention (↓intestinal IL-6, IFN-γ, TNF-α, IL-17A, ↓COX-2, Bax, PCNA, ↓NF-κB, Wnt/β-catenin, P3IK/Akt, STAT3) Modulatory effect on the Th17 response (↓CD4^+^, Rorγt^+^, IL-17^+^, IFN-γ^+^ T-cells in the lamina propria)
Khanal et al. (2011) [34]	in vitro HT-29 human colon adenocarcinoma cells in vivo chorioallantoic membrane assay	Oleocanthal	1–10 μg/mL	Antitumor effect (↑ AMPK) Apoptosis (↑ caspase-3 and poly-adenosine diphosphate-ribose polymerase, phosphorylation of p53 (Ser15)) Disruption of DNA
Bartolí et al. (2000) [35]	in vivo on rats with azoxymethane-induced CRC	n9 and n3 fatty acids (oleic and eicosapentaenoic acids)	n9: 57% of diet n3: 27.7% of diet equivalent to a 5% fat diet containing olive oil	Chemoprevention (modulation in colonic mucosa of arachidonic acid metabolism and prostaglandin E2 synthesis)
Xu et al. (2016) [36]	in vitro human CRC cell lines (SW480 and HCT15)	Apigenin	20–40 µM	↓cell proliferation (↓Wnt/β‑catenin signaling pathway)
Shao et al.(2013) [37]	in vitro human colon cancer cell lines (DLD1, HCT116, HCT8, HT29 and SW48) in vivo C.B.-17 SCID mice implanted with HCT116 cells	Apigenin	in vitro 20 mmol/L in vivo 25 mg/Kg	Synergistic effect between apigenin and ABT-263 on apoptosis (↓Mcl-1, AKT, and ERK)
Chen et al. (2018) [38]	in vitro LoVo human colon cancer cells in vivo BalbC nude mice inoculated with LoVo cells	Luteolin	IC_50_ value of 66.70 and 30.47 µmol/L at 24 and 72 h, respectively in vivo 20–40 mg/Kg	Apoptosis (↑APAF-1) Cell cycle arrest at the G2/M phase ↓tumor growth
Zuo et al. (2018) [39]	in vitro HCT116 and HT29 cells	Luteolin	-	↓CRC carcinogenesis (↑Nrf2/ARE pathway)
Reyes-Zurita et al. (2016) [40]	in vitro Caco-2 p53-Deficient Colon Adenocarcinoma Cells	Maslinic acid	IC_50_ was 40.7 ± 0.4 μg/mL IC_80_ was 56.8 μg/mL.	Apoptosis (cleavage of caspases -8 and -3, ↑t-Bid)
Reyes-Zurita et al. (2009) [41]	in vitro HT29 cells	Maslinic acid	IC_50_ was 28.8 ± 0.9 μg/mL IC_80_ was 37.5 ± 0.2 μg/mL	Apoptosis (↓Bcl-2, ↑Bax, ↑ caspase-9 and -3)
Baskar et al. (2010) [42]	in vitro human colon cancer cell lines (COLO 320 DM) in vivo Wistar rats inoculated with 1,2-dimethylhydrazine	β-sitosterol	IC_50_ was 266.2 μM in vivo10–20 mg/Kg	Chemoprevention ↓Tumor growth (↓β-catenin and PCNA)
Miene et al. (2011) [43]	in vitro human colorectal adenoma cell line LT97	3,4-dihydroxyphenylacetic acid (ES) and 3-(3,4-dihydroxyphenyl)-propionic acid (PS), metabolites of quercetin and caffeic acid, respectively.	ES: 2.5–10 µM PS: 5–25 µM	Chemoprevention after degradation of polyphenols in the gut (↑GSTT2, ↓COX2)
Losso et al. (2004) [44]	in vitro human umbilical vein endothelial cells, normal human lung fibroblast cells HEL 299, Caco-2 colon, MCF-7 breast, Hs 578T breast, and DU 145 human prostatic cancer cells	Ellagic acid	1–100 µmol/L	Anti-proliferative activity Apoptosis (↓ATP, pro-MMP-2, -9 and VEGF)

↑: Increase; ↓: Decrease; AKT: Protein kinase B; AMPK: 5′ AMP-activated protein kinase; APAF-1: Apoptotic protease activating factor 1; ATF3:Activating transcription factor 3; ATP: Adenosine triphosphate; Bax: Bcl-2-associated X protein; Bcl-2: B-cell lymphoma 2; BNIP3: BCL2 Interacting Protein 3; BNIP3L: BCL2 Interacting Protein 3 Like; Cbl: Casitas B-lineage Lymphoma; CCNB1: Cyclin B1; CCNG2: Cyclin G2; CDK: Cyclin-dependent kinase; COX2: Cyclooxygenase-2; CRC: Colorectal cancer; CYP1A1: Cytochrome P450, family 1, subfamily A, polypeptide 1; EGFR: Epidermal growth factor receptor; ERK: Extracellular-signal-regulated kinase; GSTT2: Glutathione S-transferase theta 2; HIF-1α: Hypoxia-inducible factor 1-α; HT: Hydroxytyrosol; IC_50_: half maximal inhibitory concentration; IFN-γ: Interferon-γ; IL-8: Interleukin-8; IL-17: Interleukin-17; IL-6: Interleukin-6; Mcl-1: Myeloid cell leukemia 1; NF-κB: Nuclear factor kappa-light-chain-enhancer of activated B cells; Nrf2/ARE: Nuclear factor (erythroid-derived 2)-like 2/antioxidant responsive element; OMWW: Olive mill waste water; PCNA: Proliferating cell nuclear antigen; PDCD4: Programmed cell death protein 4; PI3K: Phosphatidylinositol-3 kinase; Pro-MMP: Pro-matrix metalloproteinase; Rorγt: Retinoic acid receptor-related orphan receptor gamma thymus; STAT3: Signal transducer and activator of transcription 3; t-BiD: Truncated BH3 interacting-domain death agonist; TNF-α: Tumor necrosis factor-α; UGT1A10: UDP Glucuronosyltransferase Family 1 Member A10; VEGF: Vascular endothelial growth factor; Wnt: Wingless/Integrated.

**Table 2:** Chemical structures of principal olive oil phenols.

**Oleuropein**	**Oleocanthal**
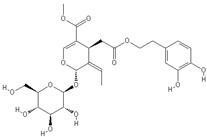	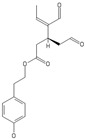
**Tyrosol**	**Hydroxytyrosol**
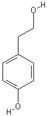	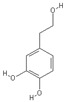
**Apigenin**	**Luteolin**
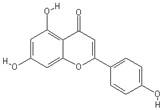	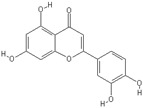
**Quercetin**	**Pinoresinol**
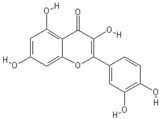	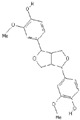
**Caffeic acid**	**Epigallocatechin-3-gallate**
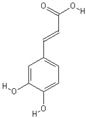	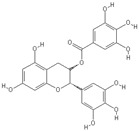
**Ellagic acid**